# Acute Coronary Syndrome: An Unusual Consequence of GERD

**DOI:** 10.1155/2015/939641

**Published:** 2015-11-24

**Authors:** Chui Man Carmen Hui, Santosh K. Padala, Michael Lavelle, Mikhail T. Torosoff, Xinjun Cindy Zhu, Mandeep S. Sidhu

**Affiliations:** ^1^Department of Medicine, Albany Medical Center, Albany, NY 12208, USA; ^2^Department of Medicine, Division of Cardiology, Albany Medical Center, Albany, NY 12208, USA; ^3^Albany Medical College, Albany, NY 12208, USA; ^4^Department of Medicine, Division of Gastroenterology, Albany Medical Center, Albany, NY 12208, USA

## Abstract

We report a case of an 83-year-old man with history of coronary artery disease and gastroesophageal reflux disease (GERD) who presented with sudden onset nocturnal dyspnea. He was diagnosed with non-ST elevation myocardial infarction based on the electrocardiographic changes and cardiac biomarker elevation. Cardiac catheterization revealed chronic three-vessel coronary artery disease, with 2 patent grafts and 2 chronically occluded grafts. While at the hospital, the patient experienced a similar episode of nocturnal dyspnea, prompting a barium esophagram, which was suggestive of a stricture in the distal esophagus from long-standing GERD. We hypothesized that he had myocardial ischemia due to increased oxygen demand from uncontrolled GERD symptoms. He had no further ischemic episodes after increasing the dose of antireflux medication over a 6-month follow-up. After presenting our case, we review the literature on this atypical presentation of GERD causing acute coronary syndrome and discuss potential mechanisms.

## 1. Introduction

Gastroesophageal reflux disease (GERD) is a common gastrointestinal disorder in the western industrial world. Although GERD classically presents with symptoms of heartburn and regurgitation of food contents, some patients may present with less typical extraesophageal cardiac or respiratory symptoms. We report an unusual case of an acute coronary syndrome in an elderly male as a consequence of GERD.

## 2. Case Report

An 83-year-old Italian male presented with sudden onset of dyspnea associated with cough and diaphoresis that woke him up from sleep at midnight. The symptoms lasted for an hour and he was taken to the hospital due to persistent discomfort. Upon presentation to the Emergency Department, he denied any chest discomfort, palpitations, dizziness, orthopnea, or lower extremity swelling. He also denied any nausea, vomiting, or epigastric discomfort. Over the previous five to six years, the patient experienced recurring episodes of nocturnal coughing and difficulty breathing during his sleep which was typically precipitated after intake of a heavy meal. Additional past medical history included extensive 3-vessel coronary artery disease (CAD) with two prior coronary artery bypass surgeries, hypertension, dyslipidemia, chronic obstructive pulmonary disease, and long-standing severe GERD. His home medications included esomeprazole, lisinopril, metoprolol succinate, aspirin, clopidogrel, and ezetimibe. Vital signs on admission revealed blood pressure of 146/95 mmHg, pulse of 90 bpm, respiratory rate of 18 per minute with 100% O_2_ saturation on 2 L of oxygen via nasal cannula. Physical exam did not reveal evidence of heart failure, wheezing, or crackles. Admission 12-lead surface electrocardiogram (ECG) revealed normal sinus rhythm with 1-2 mm horizontal ST depressions in V3 to V5, which resolved within one hour. The troponin I levels peaked at 2.6 (normal <0.04 ng/mL) and creatinine kinase levels were within normal limits.

**Figure 1:**
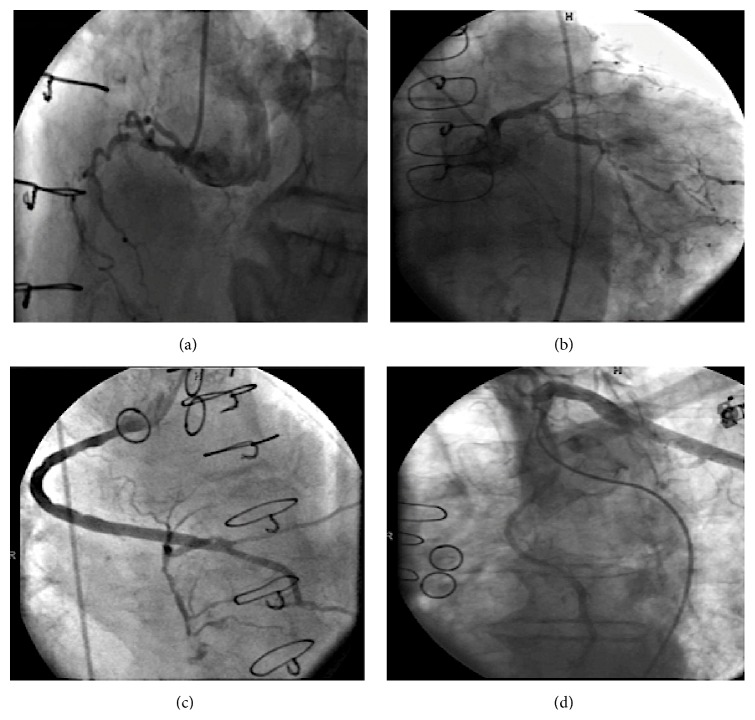
(a) Right coronary artery 100% occluded in the proximal segment. (b) Left anterior descending with 99% ostial and 100% mid occlusion and circumflex 99% distal occlusion. (c) Sequential vein graft to right posterolateral and posterior descending artery with 40% proximal disease. (d) Left internal mammary artery graft to distal left anterior descending widely patent.

Given extensive prior cardiac history, “anginal equivalent” symptoms, ischemic ECG changes, and elevated troponin I levels, non-ST elevation myocardial infarction (NSTEMI) was diagnosed and patient was started on appropriate optimal medical therapy for acute coronary syndrome. Subsequently, patient underwent an early invasive strategy of cardiac catheterization and angiography that revealed chronic, severe, native 3-vessel CAD (Figures 1(a) and 1(b)). He had patent sequential saphenous venous graft to right posterolateral and posterior descending artery and a patent left internal mammary artery to left anterior descending artery (Figures 1(c) and 1(d)). The saphenous venous grafts to the diagonal and circumflex artery were chronically occluded with evidence of collaterals. Based on the coronary anatomy the patient was managed conservatively with optimal medical therapy with no plan for percutaneous intervention or revascularization.

During the hospitalization, the patient had another episode of nocturnal dyspnea with chest tightness. He reported regurgitation and globus sensation described as “a lump in his throat with difficulty expanding his lungs.” His vital signs recorded during this episode showed an abrupt rise in blood pressure to 159/85 mmHg, heart rate to 96 bpm, and respiratory rate to 22–24 per minute, with an oxygen saturation of 98% on 2 L oxygen via nasal cannula. He denied any associated palpitations, dizziness, nausea, vomiting, or epigastric abdominal pain. ECG performed during this time showed ischemic changes, similar to his initial presentation.

**Figure 2:**
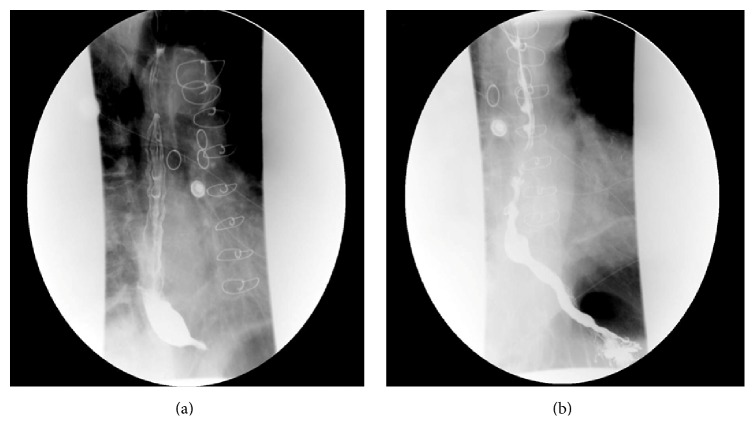
(a) Smooth short stricture in the distal esophagus slightly proximal to the gastroesophageal junction. This may represent a stricture or spasm related to reflux. (b) Multiple tertiary contractions of the distal esophagus suggestive of dysmotility.

The following day, the patient underwent a barium esophagram for evaluation of his symptoms, as an esophagogastroduodenoscopy (EGD) was deferred given recent NSTEMI. Barium esophagram demonstrated a smooth short narrowing in the distal esophagus proximal to the gastroesophageal junction, suggesting a stricture or spasm from yet controlled reflux disease (Figures 2(a) and 2(b)). In addition, he also had a flexible laryngoscopy showing normal nasopharynx, tongue, vallecula, epiglottis, and vocal cord motion. Given these findings suggesting poorly controlled reflux and the possibility of esophageal dysmotility, and temporal association of his symptoms with cardiac events, the esomeprazole dose was titrated up from 20 mg to 40 mg for symptomatic relief. The patient was stabilized with resolution of nocturnal symptoms and he was discharged home with plan to perform an outpatient upper EGD to evaluate for reflux and esophagitis. However, patient declined the elective EGD on his follow-up visit as he had no further episodes of nocturnal dyspnea on higher doses of antireflux medication. He remained symptom-free until 6-month follow-up visit.

## 3. Discussion

Gastroesophageal reflux disease (GERD) is a common gastrointestinal disorder with increasing prevalence worldwide. The prevalence of GERD ranged from 11% to 38.8% worldwide per Map of Digestive Disorders & Disease (MDD) with Mexico, Spain, Malaysia, and Yemen at the top quartile of prevalence, and Asian countries in the lowest quartile [1]. In USA, approximately 7 million people are affected [2]. According to National Digestive Disease Information Clearinghouse (NDDIC), 20% of the population had reflux symptoms at least once a week in 2004; 8.9 million ambulatory visits in 2009 and 4.7 million hospitalizations in 2010 were attributed to GERD [3]. Furthermore, the prevalence of GERD in patients with CAD is higher, with some studies reporting prevalence ranging from 40% to 78% [4].

GERD is caused by an impaired antireflux barrier and defective lower esophageal sphincter, leading to reflux of gastric acid into the esophagus. Typical GERD symptoms are heartburn and regurgitation of food contents. However, many patients with GERD may present with extraesophageal symptoms such as chest pain or discomfort mimicking angina, chronic cough, wheezing, dyspnea, globus sensation, hoarseness, or recurrent pneumonia as their primary presentation [5–7]. Identifying the cause and effect relationship between respiratory symptoms and GERD has been a clinical challenge. Two mechanisms have been proposed to be responsible for respiratory symptoms induced by gastric reflux: (1) vagal reflex response from stimulation of the vagus nerve by gastric acidic content, resulting in bronchoconstriction and (2) microaspiration of gastric contents causing direct irritation or trauma to the upper airway [5–7]. In a single-center study by Salvador et al., 30 patients with GERD underwent simultaneous 24-hour multichannel intraluminal impedance pH monitoring and continuous O_2_ saturation monitoring via pulse oximetry [8]. Approximately 60% of the reflux episodes were associated with oxygen desaturation. Furthermore, the high prevalence of O_2_ desaturation was found mostly in GERD patients with primary respiratory complaints [8].

We present a case of an atypical presentation of GERD leading to NSTEMI, likely from demand ischemia in the setting of known severe 3-vessel native CAD as well as chronic total occlusions of venous grafts. Given the patient's extensive cardiac history and limited cardiac reserve, the physiologic response of elevated blood pressure, heart rate, respiratory rate, and transient hypoxia was likely significant enough to cause myocardial ischemia and injury. GERD may also lead to demand ischemia and cause NSTEMI through other mechanisms. It is well known that pain can cause an increase in myocardial oxygen demand through enhanced adrenergic activity with increased heart rate and blood pressure [9]. Pain from esophageal spasm is one distinct possibility for precipitating ischemia in this patient.

It is also possible that myocardial ischemia in our patient was due to “esophagocardiac reflex,” which describes myocardial ischemia associated with chemical esophageal stimulation. Chauhan et al. demonstrated that esophageal acid stimulation in patients with documented CAD on angiogram resulted in typical chest discomfort and a significant reduction in coronary blood flow as measured by intracoronary Doppler in 9 of 14 (64%) patients [10]. In a study by Dobrzycki et al., 50 patients with angiographically proven CAD underwent simultaneous continuous ECG and esophageal pH monitoring for 24 hours to assess for ST-segment depression episodes and total duration of ischemic episodes [11]. Of 218 episodes of ST-segment depression, 45 (20.6%) correlated with pathologic reflux. Compared to patients without GERD, patients with GERD were found to have significantly higher number of ST-segment depression episodes and total ischemic burden. The authors also demonstrated significant improvement in ST-segment depression episodes and total ischemic burden following a 7-day course of proton pump therapy (PPI) in patients with GERD suggesting that restoring normal esophageal pH might eliminate acid-derived esophagocardiac reflex and hence myocardial ischemia [11]. Liu et al. reported similar findings of longer duration and higher incidence of ischemic events in patients with CAD and gastric reflux [12]. Short course of PPI not only resulted in fewer ischemic events, but also significantly improved the general health-related quality of life of patients [12]. Furthermore, Swiatowski et al. demonstrated that 14 days of PPI therapy in 34 patients with GERD and CAD caused a significant increase in the amount of time before maximal ST depression occurred during exercise stress test, showing that PPI therapy has a favorable effect on cardiac reserve [13].

For our patient, high dose of PPI was initiated to control his reflux symptoms along with further optimization of medical therapy for his CAD in order to augment efforts at secondary prevention of future ischemic events. Medication adjustment resulted in resolution of nocturnal symptoms, which were likely a manifestation of GERD and angina.

In conclusion, there is a high prevalence of GERD in patients with CAD. It is underappreciated that GERD can potentially cause myocardial ischemia by increasing myocardial oxygen demand or by decreasing myocardial oxygen supply (esophagocardiac reflex). Thus, it is critically important to recognize this association and initiate treatment with PPIs in appropriate patients with CAD and concomitant GERD as it might improve GERD and prevent future adverse cardiac events.

## References

[1] Guarner F., Lazaro, Gascon, Royo, Eximan, Herrero (2008). *Map of Digestive Disorders and Diseases*.

[2] University of Florida (2012). *Gastroesophageal Reflux Disease*.

[3] http://www.niddk.nih.gov/health-information/health-statistics/Pages/digestive-diseases-statistics-for-the-united-states.aspx.

[4] Liuzzo J. P., Ambrose J. A. (2005). Chest pain from gastroesophageal reflux disease in patients with coronary artery disease. *Cardiology in Review*.

[5] Gurski R. R., Pereira Da Rosa A. R., Do Valle E., De Borba M. A., Valiati A. A. (2006). Extraesophageal manifestations of gastroesophageal reflux disease. *Jornal Brasileiro de Pneumologia*.

[6] DeVault K. R. (2003). Extraesophageal symptoms of GERD. *Cleveland Clinic Journal of Medicine*.

[7] Irwin R. S., Madison J. M. (2002). Diagnosis and treatment of chronic cough due to gastro-esophageal reflux disease and postnasal drip syndrome. *Pulmonary Pharmacology & Therapeutics*.

[8] Salvador R., Watson T. J., Herbella F. (2009). Association of gastroesophageal reflux and O_2_ desaturation: a novel study of simultaneous 24-h MII-pH and continuous pulse oximetry. *Journal of Gastrointestinal Surgery*.

[9] Cousins M. J., Bridenbaugh P. O., Carr D. B., Horlocker T. T., Wu C. L., Liu S. S. (2009). Neural blockade: impact on outcome. *Cousins and Bridenbaugh’s Neural Blockade in Clinical Anesthesia and Pain Medicine*.

[10] Chauhan A., Mullins P. A., Taylor G., Petch M. C., Schofield P. M. (1996). Cardioesophageal reflex: a mechanism for ‘linked angina’ in patients with angiographically proven coronary artery disease. *Journal of the American College of Cardiology*.

[11] Dobrzycki S., Baniukiewicz A., Korecki J. (2005). Does gastro-esophageal reflux provoke the myocardial ischemia in patients with CAD?. *International Journal of Cardiology*.

[12] Liu Y., He S., Chen Y. (2013). Acid reflux in patients with coronary artery disease and refractory chest pain. *Internal Medicine*.

[13] Swiatowski M., Jacek B., Klopocka M. (2004). Suppression of gastric acid production may improve the course of angina pectoris and the results of treadmill stress test in patients with coronary artery disease. *Medical Science Monitor*.

